# Investigation of a Magnetic Tunnel Junction Based Sensor for the Detection of Defects in Reinforced Concrete at High Lift-Off

**DOI:** 10.3390/s19214718

**Published:** 2019-10-30

**Authors:** Muhamad Arif Ihsan Mohd Noor Sam, Zhenhu Jin, Mikihiko Oogane, Yasuo Ando

**Affiliations:** 1 Department of Applied Physics, Tohoku University, Sendai 980-8579, Japan; zjin@mlab.apph.tohoku.ac.jp (Z.J.); oogane@mlab.apph.tohoku.ac.jp (M.O.); ando@mlab.apph.tohoku.ac.jp (Y.A.); 2 Graduate Program in Spintronics, Tohoku University, Sendai 980-8578, Japan; 3 Center for Science and Innovation in Spintronics (Core Research Cluster), Organization for Advanced Studies, Tohoku University, Sendai 980-8577, Japan; 4 Center for Spintronics Research Network, Tohoku University, Sendai 980-8577, Japan

**Keywords:** non-destructive testing, reinforced concrete, lift-off, magnetic tunnel junction sensors

## Abstract

Magnetic flux leakage (MFL) testing is a method of non-destructive testing (NDT), whereby the material is magnetized, and when a defect is present, the magnetic flux lines break out of the material. The magnitude of the leaked magnetic flux decreases as the lift-off (distance from the material) increases. Therefore, for detection at high lift-off, a sensitive magnetic sensor is required. To increase the output sensitivity, this paper proposes the application of magnetic tunnel junction (MTJ) sensors in a bridge circuit for the NDT of reinforced concrete at high lift-off. MTJ sensors were connected to a full-bridge circuit, where one side of the arm has two MTJ sensors connected in series, and the other contains a resistor and a variable resistor. Their responses towards a bias magnetic field were measured, and, based on the results, the sensor circuit sensitivity was 0.135 mV/mT. Finally, a reinforced concrete specimen with a 1 cm gap in the center was detected. The sensor module (with an amplifier and low pass filter circuits) could determine the gap even at 50 cm, suggesting that MTJ sensors have the potential to detect defects at high lift-off values and have a promising future in the field of NDT.

## 1. Introduction

Non-destructive testing (NDT) is the evaluation of a specimen without causing damage. One of the applied areas of NDT is in the testing of reinforced concrete, which is one of the most popular materials in the world and is used in many structures, such as buildings and bridges. Although it increases the structural integrity of structures, it is still susceptible to corrosion and breaks when exposed to harsh environments or an excessive amount of force [1,2]. Therefore, the management and maintenance of these structures by NDT is important to prevent them from collapsing, which can result in the loss of life and environmental damage. There are several methods for the NDT of reinforced concrete [3], with ultrasonic testing (UT) [4] and X-ray diffraction (XRD) [5] being the most popular methods. However, both of these methods are time-consuming or need expensive equipment to determine corrosion in the depth of the reinforced concrete.

Since the steel bars used for the reinforced concrete are made of a type of ferromagnetic material, defects like cracks and corrosion can change the structure of magnetic domains, and their macro-properties are also changed. Taking advantage of these properties, magnetic flux leakage (MFL) testing [6,7] is considered for its application. In principle, the steel rebar is magnetized, and due to the changes in the magnetic properties caused by a defect, magnetic flux lines break out of the steel rebar. The magnitude of the leaked magnetic flux is dependent on the shape and size of the defect. In order to detect these leaks, a sensitive magnetic sensor is required. Another factor that needs to be considered is the effect of the lift-off (the distance between the sensor and the steel rebar) on the detection of the defects. Most studies focus on detection at low lift-off values, where the leaked magnetic flux is the strongest, thereby making the detection more accurate [8]. However, in some cases, the steel rebar is located further away from the concrete’s surface, resulting in the magnitude of the leaked magnetic flux being very small, making detection difficult.

In conventional MFL, Hall effect sensors are commonly used to detect the leaked magnetic flux. However, these sensors have a very low sensitivity range, which may cause difficulties when performing measurements at high lift-off. Therefore, magnetic tunnel junction (MTJ) based sensors have attracted massive attention due to their high sensitivity and low detectivity range [9,10]. In contrast to other available magnetic sensors on the market, such as Hall sensors, magneto-impedance sensors, and inductive coils, the sizes of MTJ sensors can be miniaturized. Moreover, the sensitivity of MTJ sensors is independent of field frequency [11]. Due to these excellent properties, MTJ sensors have not only attracted attention in the field of bio-magnetic sensors [12] but have also become one of the main prospects in non-destructive testing sensors [13,14].

Studies on MTJ sensors focus on finding the suitable free layer structure of the MTJ film stack [15,16] or optimizing the integration of the MTJ sensor [17] in order to increase the sensitivity of the sensor and reduce noise. However, several reports have mentioned that by integrating the MTJ sensors in bridge circuits, the sensitivity of the overall system was improved [18,19]. Other factors, such as the sensor circuit (amplifier circuit and filter circuit) also play an important role in producing a better-quality response. Therefore, this research will focus on improving the overall sensor output by implementing two MTJ sensors in a full-bridge circuit, followed by an amplifier circuit and a low pass filter to detect defects at high lift-off.

This paper will investigate the ability of two MTJ sensors in one arm of a full-bridge circuit to detect defects in the steel rebar at high lift-off values. First, two MTJ sensors were chosen and connected to one arm of a full-bridge circuit to increase the overall sensitivity. The response of the MTJ sensor bridge circuit was measured in a magnetic shield room by applying a bias magnetic field. Finally, using the constructed system, the measurement of a concrete specimen, which contains cracked steel rebar, was conducted with varying different lift-off values to determine the system’s ability to detect defects at large lift-off values.

## 2. Materials and Methods

**Figure 1:**
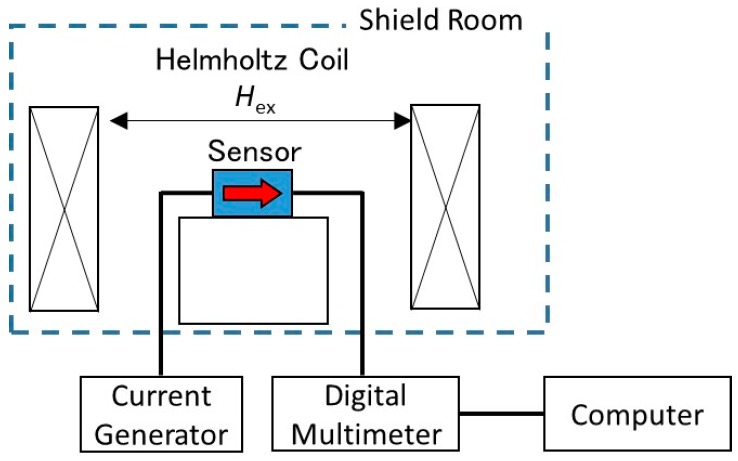
Experiment setup for the magnetic shield room measurement.

The MTJ sensors used were fabricated into the following structure: Substrate/Ta (5)/NiFe (70)/Ru (0.9)/CoFeB (3)/MgO (1.64)/CoFeB (3)/Ru (0.9)/CoFe (5)/IrMn (10)/Ta (5)/Ru (50) (the thicknesses shown in parentheses are in nanometers). The magnetization direction of the free layer and pinned layer are set orthogonally to each other. One sensor unit consists of 1740 MTJ structures (870 connected in series and 2 connected in parallel) [20,21]. The sensors were then connected in a full-bridge circuit, where one arm contains the two MTJ sensors (MTJ①’s sensitivity is facing the +*y* direction and the MTJ②’s sensitivity is facing the −*y* direction), while the opposite arm contains a variable resistor and a chip resistor. This means that when the magnetic flux is in the +*y* direction, the resistance in MTJ① will decrease, while the resistance in MTJ② will increase. The variable resistor functions to control the offset of the voltage output before every measurement was taken (the offset was set to 0 V at the start), and the chip resistor value was similar to that of the MTJ sensors (430 Ω). This setup ensured that each measurement started at 0 V, so the output voltage change corresponding to the leaked magnetic flux could be clearly seen. The bridge circuit’s sensor response was determined by applying a bias magnetic field using a Helmholtz coil and recording the resulting resistance value. A schematic diagram of the measurement setup is shown in Figure 1.

**Figure 2:**
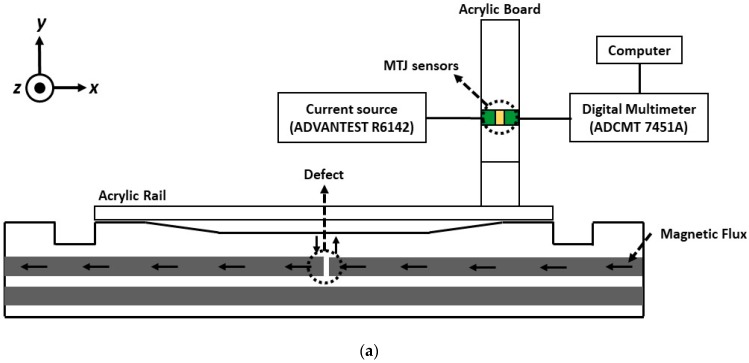

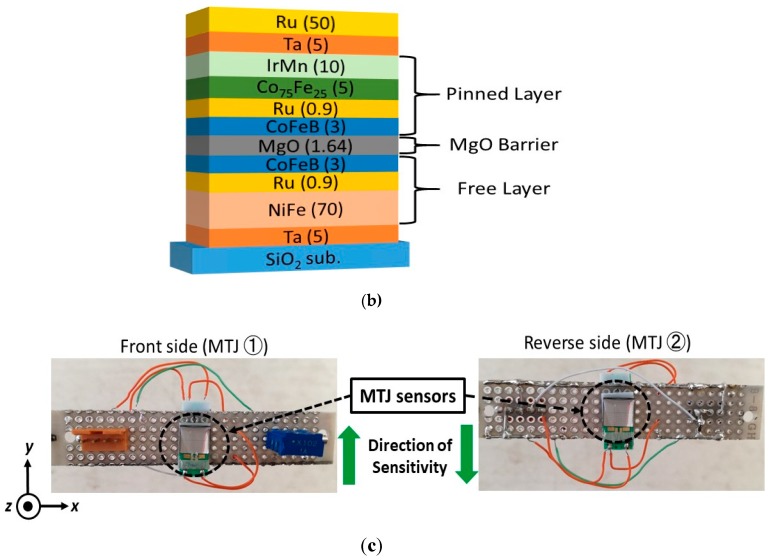
(**a**) The reinforced concrete measurement setup. (**b**) Schematic diagram of the magnetic tunnel junction (MTJ) sensor. (**c**) MTJ sensor setup, the front side sensor (MTJ①) is placed right side up, resulting in the sensor’s sensitive direction facing the +*y* direction, while the reverse side sensor (MTJ②) is placed upside down, resulting in the sensor’s sensitive direction facing the −*y* direction.

The signal from the MTJ sensor circuit was then passed through an amplifier circuit where the signal was amplified 1000 times and a low pass filter with a cut-off frequency of 10 Hz was used. The schematic diagram for the measurement of the concrete specimen is shown in Figure 2. The current source provides a bias DC current to the sensor. The magnetic flux will cause a change in the resistance of the sensor, so the resulting output voltage will also fluctuate. A digital multimeter recorded the output voltage, and the data were observed on a computer, where the presence and location of the defect could be analyzed.

The concrete specimen was magnetized using a permanent magnet (neodymium magnet, magnetic strength = 0.7 T), magnetized in the *x* direction. The magnetization process was done once as once the steel rebar is magnetized, it does not lose its magnetism. An acrylic rail placed on the concrete’s surface acts as a measurement platform and guide for the sensor circuit. The sensor was placed on an acrylic board attached to a push car, allowing it to slide on the rail smoothly. For measurements at different lift-off distances, the sensor was moved to the appropriate lift-off height on the acrylic board. The sensor is moved along the *x*-axis and the normal component of the magnetic flux (*y*-axis) was measured.

## 3. Results and Discussion

### 3.1. Circuit Response

**Figure 3:**
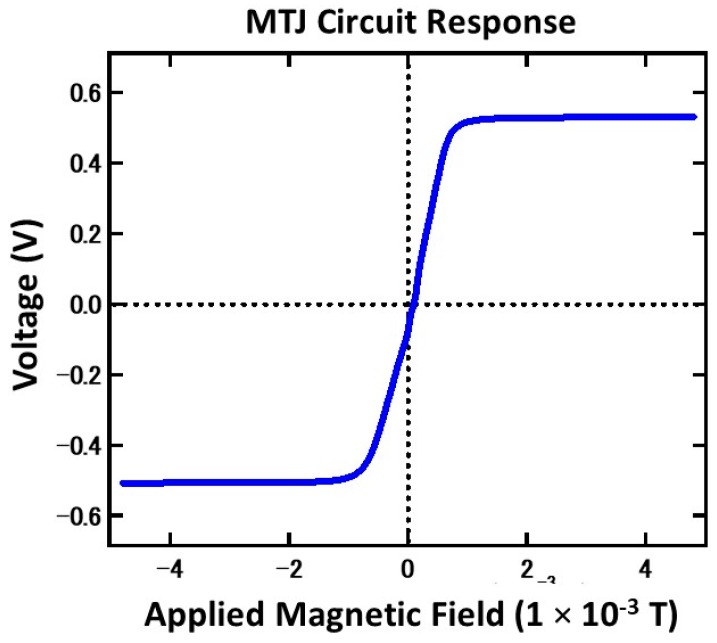
MTJ bridge circuit response towards an applied magnetic field inside a magnetic shield room.

Before moving on to real specimen measurement, the constructed circuit response was measured to determine its response when a bias magnetic field is applied. The measurement was conducted in a magnetic shield room to prevent any disturbance to the sensors during measurement. The MTJ bridge circuit was placed in a Helmholtz coil, which applied a bias magnetic field of −4.8 mT to 4.8 mT, and a current of 6.0 mA was used to power the sensor. A small bias magnetic field was used, since the target magnetic range was small. The resulting circuit response is shown in Figure 3. The sensitivity could be determined by taking the derivation of the linear response.

From the observed results, the MTJ bridge circuit showed a normal linear magnetic response with a small offset (71.4 mV). The maximum sensitivity of the circuit, taken from the linear derivative, is 0.135 mV/mT in the most sensitive region (1.44 × 10^−1^–1.6 × 10^−2^ mT). Due to restrictions in the measurement setup, the values in the lower magnetic field were difficult to obtain. Nevertheless, in the range of 0–1.6 × 10^−2^ mT, a sensitivity of 0.112 mV/mT was obtained.

### 3.2. Concrete Specimen Measurement

**Figure 4:**
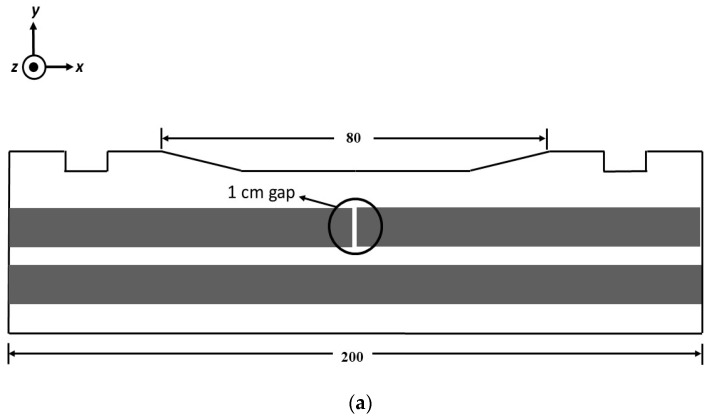

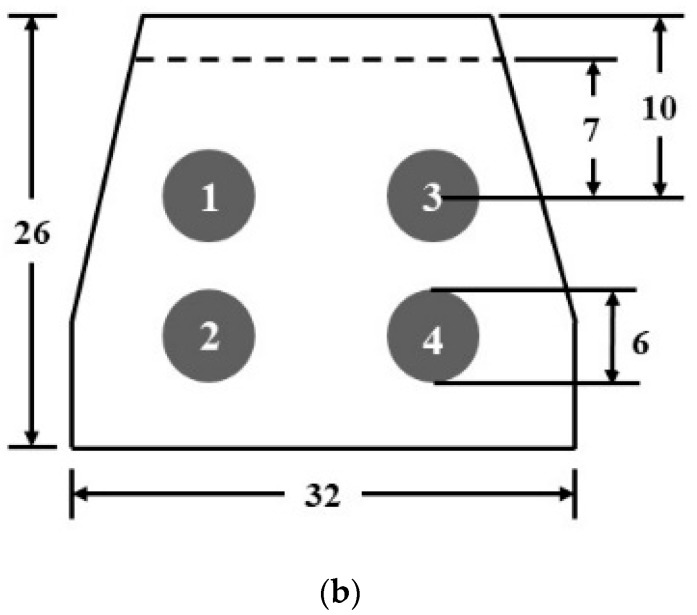
(**a**) Side view of the concrete specimen. (**b**) Top view of the concrete specimen. Only steel rebar 1 is measured. (All units are in centimeters).

**Figure 5:**
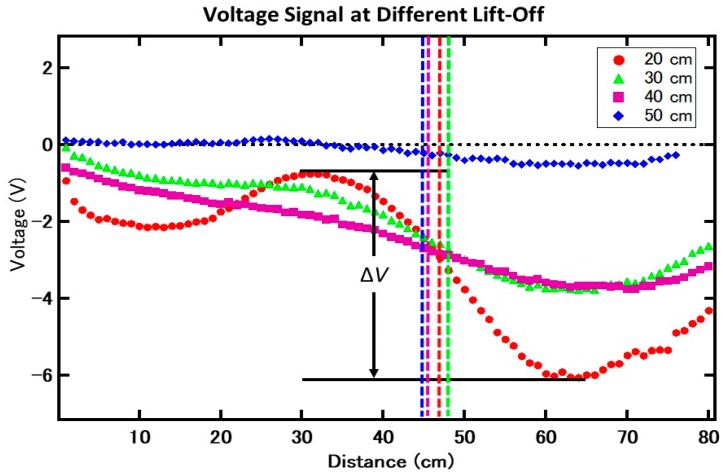
Results of the output voltage signal at each respective voltage. The dotted lines show the estimated defect location at every lift-off, corresponding to their respective graph colors. Δ*V* is the voltage difference between the highest peak and lowest peak for each measurement.

In order to determine the constructed system performance, a reinforced concrete specimen was measured. The schematic diagram of the concrete specimen is shown in Figure 4. In this work, only steel rebar 1, which has a defect (a gap of 1 cm in the center of the concrete), was measured. Since the crack location was already known, the measurement range was set to 80 cm, with the crack at the 47 cm mark. The sensor push car was slid across the rail by hand every 1 cm, and the output voltage was recorded 50 times in order to further reduce the noise affecting the final signal. The results for the final signal output for each lift-off are shown in Figure 5. The difference between the peak and trough (Δ*V*) and the estimated crack location was analyzed from the resulting output voltage.

**Figure 6:**
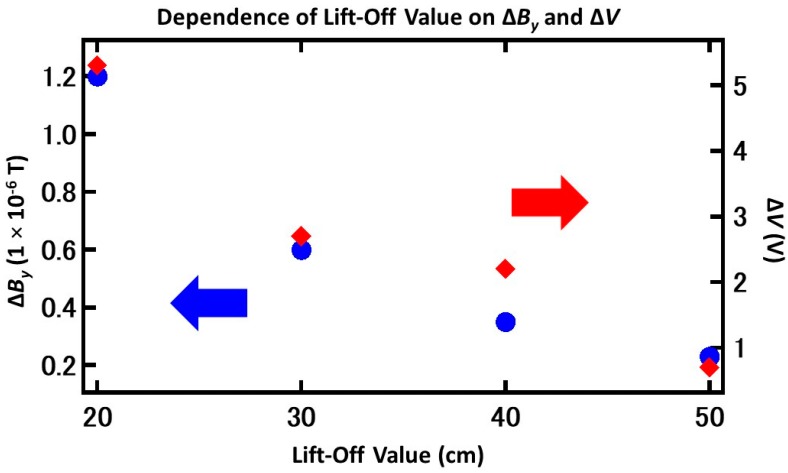
Results of the calculated Δ*B_y_* (difference between the highest peak and lowest peak from the theoretical calculation based on Equation (1)) and Δ*V* showed similar trends as lift-off increased.

The observed trend from the results is that the Δ*V* decreased as lift-off increased, which is similar to the calculation using the magnetic dipole theory [22], as shown in Figure 6. By converting *H_y_* into *B_y_*, the value of Δ*B_y_* was determined:

(1) $$H_{y}=-m_{1}\frac{x}{x^{2}+{\left(y+h\right)}^{2}}+m_{2}\frac{x}{x^{2}+{\left(y+h+d\right)}^{2}}$$

(2) $$m_{1}=H_{0}\frac{\left(2d+w\right)}{2\pi }{\left\{1-{\left(\frac{\mu -\mu_{0}}{\mu +\mu_{0}}\right)}^{2}{\left(\frac{w}{4h}\right)}^{2}\right\}}^{-1}$$

(3) $$m_{2}=H_{0}\frac{\left(2d+w\right)}{2\pi }{\left\{1-{\left(\frac{\mu -\mu_{0}}{\mu +\mu_{0}}\right)}^{2}{\left(\frac{w}{2\left(xh+2d\right)}\right)}^{2}\right\}}^{-1}$$

where *h* is the distance from the surface of the concrete to the top of the steel rebar; *w* is the width of the defect; *d* is the depth of the defect; *m*_1_ and *m*_2_ are the dipole moment per unit length for each dipole; and *μ* and *μ*_0_ are the permeabilities of the magnetic material and air, respectively.

**Figure 7:**
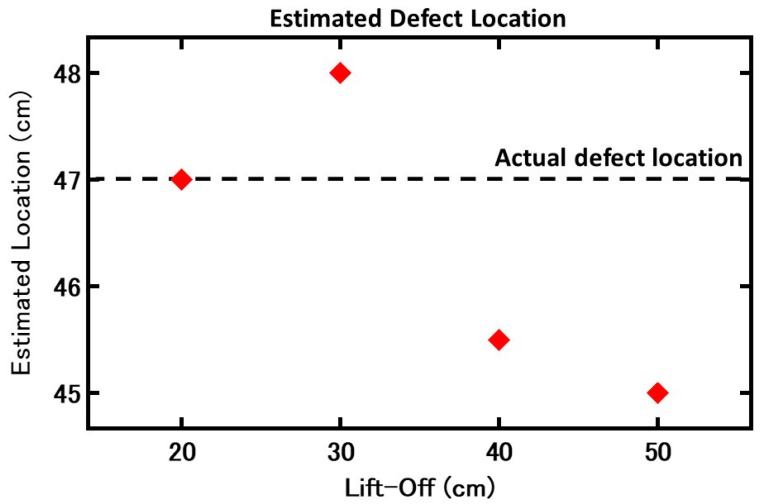
Estimated defect location based on the results. A loss in accuracy occurs as lift-off increases.

However, as seen in Figure 7, to estimate the crack location as the lift-off increases, the crack location slightly shifts. The reason for this phenomenon is still unclear, but we believe it was caused by the magnetic flux distribution from a defect or environmental magnetic noise.

In summary, an MFL measurement system consisting of two MTJ sensors in a full bridge circuit configuration was constructed. This system demonstrated similar behavior to the theoretical calculation, whereby the magnitude of the leaked magnetic flux decreases as the lift-off increases. Based on the results, the system showed the capability to detect the defects (cracks) in reinforced concrete at high lift-off values.

## 4. Conclusions

In this work, two MTJ sensors (connected in series) in one arm of a full-bridge circuit were designed and developed for the NDT of the reinforced concrete at high lift-off values. In a controlled environment, the system showed a good linear response, and, based on the measurement of a real concrete specimen, the system was able to identify the presence of the crack even at high lift-off values (50 cm). However, from the results, the estimated location of the crack slightly shifted as the lift-off height increased, resulting in a decrease of accuracy. Nevertheless, the results showed the capability of MTJ sensors in NDT to detect and locate defects in reinforced concrete structures even at high lift-off values (50 cm). In order to realize real-field measurements, future work will include increasing the accuracy of the sensing system, speed, and efficiency of the device. Specimens with various defect dimensions will also be tested to further determine the system’s possible practical applications.
